# Chromogenic hydroxyanthraquinone-based enzyme substrates for the detection of microbial β-d-galactosidase, β-d-glucuronidase and β-d-ribosidase†

**DOI:** 10.1039/d4ra06418f

**Published:** 2025-02-07

**Authors:** Michael Burton, Amy Garcha, Emma C. L. Marrs, John D. Perry, Stephen P. Stanforth, Graeme Turnbull, Hayley J. Turner

**Affiliations:** a Glycosynth Ltd 14 Craven Court, Winwick Quay, Cheshire Warrington WA2 8QU UK g.turnbull@northumbria.ac.uk; b Department of Microbiology, Freeman Hospital Newcastle upon Tyne NE7 7DN UK; c Department of Applied Sciences, Northumbria University Newcastle upon Tyne NE1 8ST UK

## Abstract

Di-β-d-galactopyranoside derivatives of quinizarin (1,4-dihydroxyanthraquinone) and anthrarufin (1,5-dihydroxyanthraquinone) were evaluated as microbial enzyme substrates in Columbia agar medium for the detection of clinically important microorganisms. Furthermore, these substrates were evaluated both in the presence and absence of iron salts which could chelate to the aglycone after microbial hydrolysis of the substrate. The quinizarin-based substrate resulted in the formation of black microbial colonies in the presence of iron salts and orange colonies in their absence. In contrast, yellow-coloured microbial colonies were observed with the anthrarufin-based substrate regardless of whether iron salts were present or not. 1-Hydroxyanthraquinone-β-d-galactopyranoside also resulted in yellow-coloured microbial colonies in the absence of iron salts and an extended study of this substrate using 38 clinical strains of *E. coli* indicated its potential for identifying this microorganism when compared to a commercially available indoxyl based substrate. 1-Hydroxyanthraquinone-β-d-glucopyranuronide was also evaluated for *E. coli* detection, but this substrate was deemed less effective than its indoxyl-based counterpart. 1-Hydroxyanthraquinone-β-d-ribofuranoside was evaluated for its potential to detect *Pseudomonas aeruginosa* and this substrate shows promise for this application.

## Abstract 2

Glycoside derivatives of quinizarin, anthrarufin, and 1-hydroxyanthraquinone for use in microbial diagnostics.

## Graphical abstract

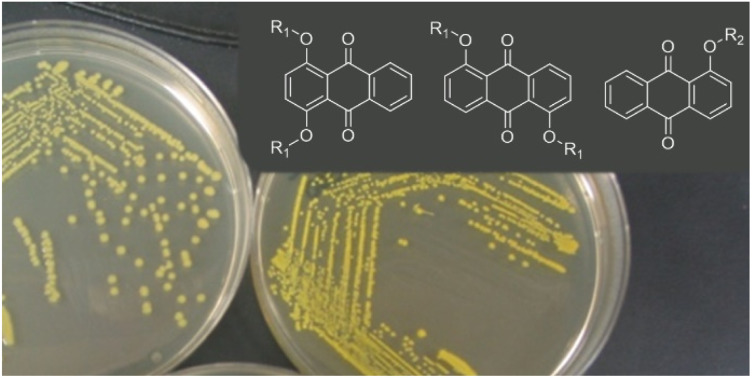

## Introduction

**Fig. 1:**
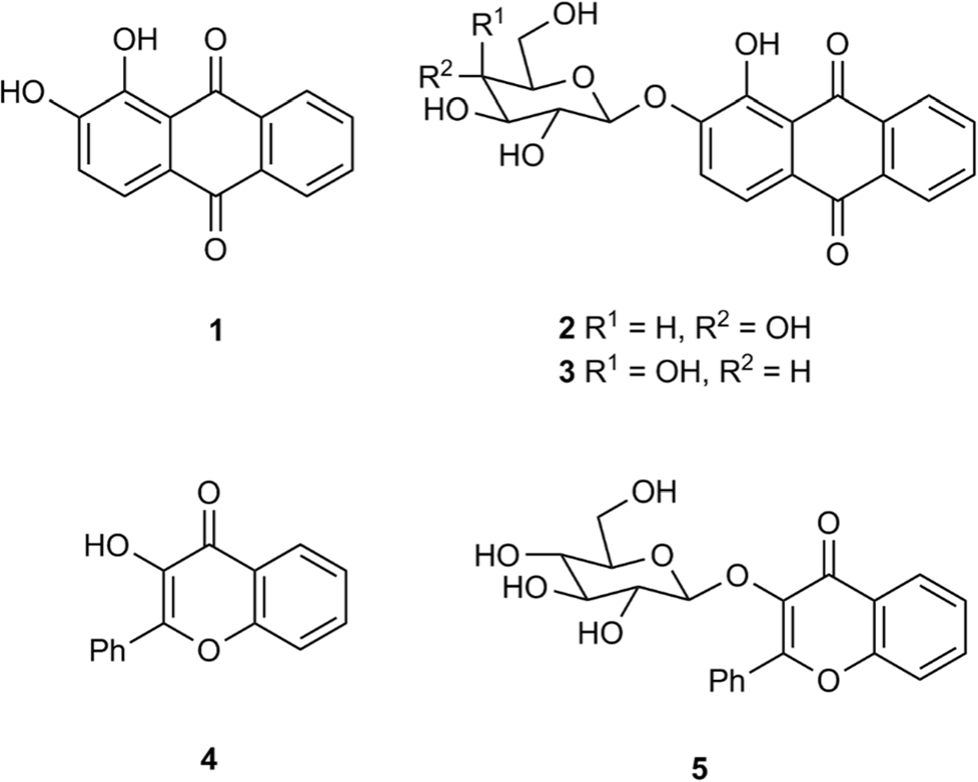
Glycosides of alizarin 1 and 3-hydroxyflavone 4.

The application of chromogenic glycosidase enzyme substrates (Ar–O–sugar; Ar = aromatic group) in bacteriological culture media for the detection of microbiological species (or groups of species) is well established.^1–4^ If an appropriate glycosidase is produced by the microorganism of interest, the substrate can undergo hydrolytic cleavage thus liberating the sugar moiety from its phenolic aglycone (Ar–OH). The aglycone may be coloured, *e.g. ortho*-nitrophenyl-β-d-galactopyranoside produces the yellow-coloured *ortho*-nitrophenol, and hence the presence of a β-galactosidase-producing microorganism can be inferred.^5^ If the aglycone is only weakly coloured, then a secondary reaction to develop a colour can be employed, *e.g.* in the presence of metal ions, the hydrolysis of the anthraquinone derivatives alizarin-β-d-glucopyranoside 2 and alizarin-β-d-galactopyranoside 3 (Fig. 1) produces alizarin 1 which subsequently forms strongly coloured metal chelates (purple with Fe(iii) ions and red with Al(iii) ions) through chelation of alizarin's catechol moiety with the metal ion.^6,7^ Other chromogenic catechol-based sugar derivatives which similarly rely on metal ion chelation to produce colour have also been used successfully in diagnostic microbiology *e.g.* glycosides of dihydroxycoumarins,^6^ 3′,4′-dihydroxyflavone^6^ and 2,3-dihydroxynaphthalene.^8^ Although not a catechol derivative, 3-hydroxyflavone-β-d-glucopyranoside 5 has been shown to produce brown colonies (presumably resulting from chelation involving both the hydroxy and carbonyl groups of 3-hydroxyflavone 4) from a limited number of bacteria in the presence of Fe(iii) ions (Fig. 1).^6^

**Fig. 2:**
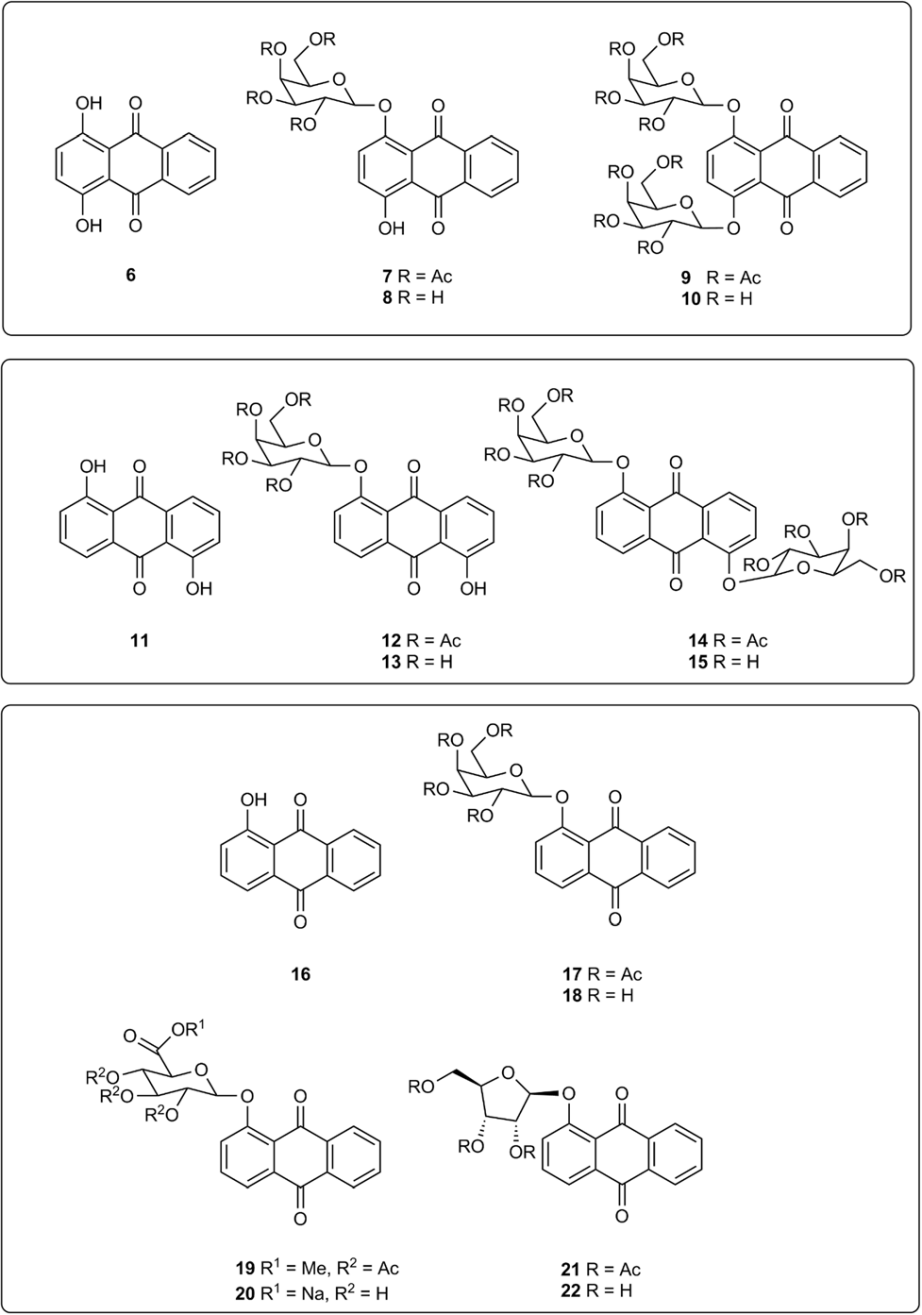
Glycosidase substrates and their associated aglycones.

The alizarin glycosides 2 and 3 were shown to be highly sensitive chromogenic substrates for the detection of bacterial β-d-glucosidase and β-d-galactosidase respectively in Columbia agar medium supplemented with metal ions. Additionally, the insoluble coloured chelate formed after hydrolysis of these substrates remained tightly localised on bacterial colonies. This allows microorganisms producing the enzyme of interest to be clearly differentiated from those that do not within polymicrobial cultures when the detection of specific microorganisms is required.^9^ In view of the success of these two substrates, it was of interest to examine other hydroxyanthraquinone-based substrates and hence derivatives of quinizarin 6, anthrarufin 11, and 1-hydroxyanthraquinone 16 were selected as the core aglycones for study (Fig. 2). Although these three compounds are not catechols, potential metal ion chelation could occur between a hydroxy group and the adjacent carbonyl group as noted above for substrate 4. β-d-Galactopyranoside derivatives of these aglycones were initially chosen as target substrates because of their potential for detecting pathogenic bacteria within the coliform group *Enterobacterales*.^10^ Quinizarin mono-β-d-galactopyranoside 8 and quinizarin di-β-d-galactopyranoside 10 have been reported previously as potential antitumor agents.^11^ The anthrarufin-β-d-galactopyranoside derivatives 13 and 15 and 1-hydroxyanthraquinone-β-d-galactopyranoside 18 have not been previously reported.

## Experimental

Quinizarin 6 was subjected to a Michael-type glycosylation reaction with an excess of acetobromo-α-d-galactopyranose (AB-Gal) under basic, phase transfer conditions (see ESI†) producing a mixture of the mono- and di-sugar derivatives 7 (4.5% yield) and 9 (19% yield) respectively. Zemplén deprotection (cat. NaOMe in MeOH) of compound 9 afforded the required substrate 10 (97% yield). Anthrarufin 11 similarly afforded a mixture of mono- and di-sugar derivatives 12 (17% yield) and 14 (12% yield) respectively with the latter compound yielding the substrate 15 (95% yield) after deprotection. 1-Hydroxyanthraquinone 16 and AB-Gal produced compound 17 (30% yield) which upon deprotection gave the substrate 18 (84% yield). The methyl ester of 1,2,3,4-tetra-*O*-acetyl β-d-glucopyranuronic acid and compound 16 were reacted under basic conditions in the presence of Ag_2_CO_3_ producing the methyl ester 19 (33% yield). Hydrolysis of this ester by NaOH in acetone yielded substrate 20 (20% yield). 1-Hydroxyanthraquinone 16 was reacted with 1-trichloroacetimidyl-2,3,5-triacetyl-β-d-ribofuranose in the presence of BF_3_ etherate giving the protected riboside 21 in low (1.7%) yield. Unreacted 1-hydroxyanthraquinone 16 was present in the reaction mixture but all the 1-trichloroacetimidyl-2,3,5-triacetyl-β-d-ribofuranose had been consumed. Substrate 22 was obtained (81% yield) by Zemplén deprotection of compound 21.

### Microbiology evaluation

For each substrate, 100 mL of molten Columbia agar (Thermo Fisher, Basingstoke, UK) was prepared according to manufacturer's instructions and supplemented with 50 mg of ferric ammonium citrate (FAC) before sterilization by autoclaving at 116 °C for 20 minutes. The agar was then held at 50 °C in a water bath prior to dispensing. 30 mg of each anthraquinone-based substrate was weighed into a sterile glass bottle and dissolved in a minimum volume of 1-methyl-2-pyrrolidone (up to a maximum of 0.5 mL). Each dissolved substrate was then added to molten Columbia agar and gently mixed before dispensing 20 mL volumes into five sterile Petri dishes. The plates were dried in a hot room for 10 minutes to remove surface moisture and then stored at 4 °C prior to inoculation. The final substrate concentration was 300 mg L^−1^. For the β-d-galactosidase substrates, and unless otherwise stated in the text, isopropyl β-d-thiogalactopyranoside (IPTG; 30 mg L^−1^) was added to the medium as an inducer of β-galactosidase activity.^12^ For the β-d-glucuronidase 20 and β-d-ribosidase 22 substrates, no IPTG or FAC was added to the medium. Substrate-free control plates were prepared containing 500 mg per L FAC, with and without 0.5% 1-methyl-2-pyrrolidone to examine the impact of solvent on growth.

**Fig. 3:**
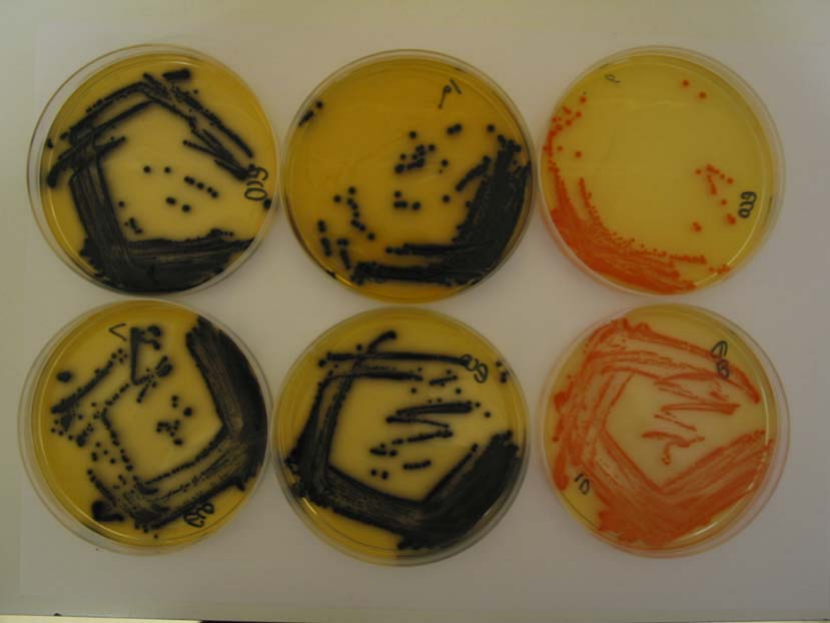
*E. coli* (NCTC 10418) and substrate 10. Top left, with FAC and IPTG; bottom left, with iron(ii) acetylacetonate and IPTG; top middle, with iron(ii) acetate and IPTG; bottom middle with iron(iii) acetylacetonate; top right, without FAC and with IPTG; bottom right, without FAC and IPTG.

**Fig. 4:**
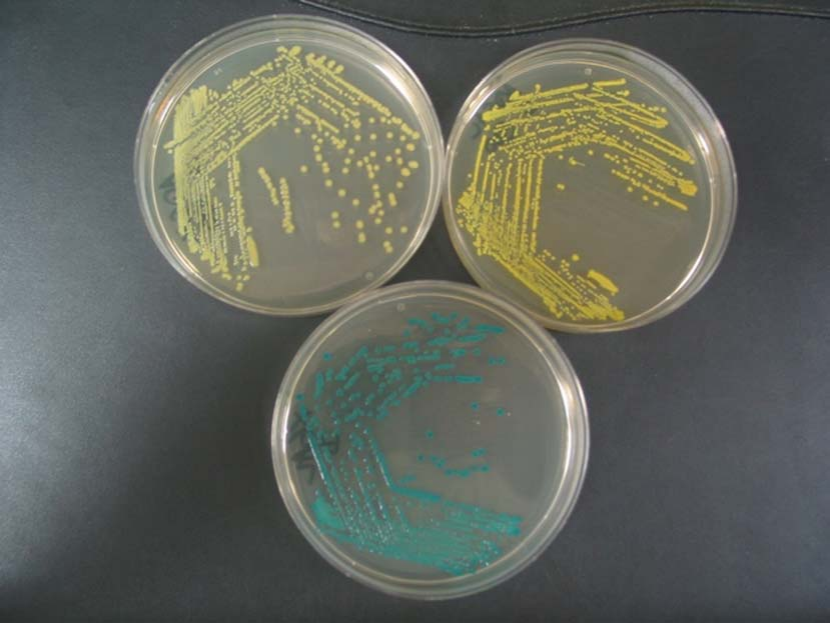
*E. coli* (NCTC 12241) with substrates 18 and X-Gal. Top left, substrate 18 (100 mg L^−1^); top right, substrate 18 (300 mg L^−1^); bottom, X-Gal (80 mg L^−1^).

**Fig. 5:**
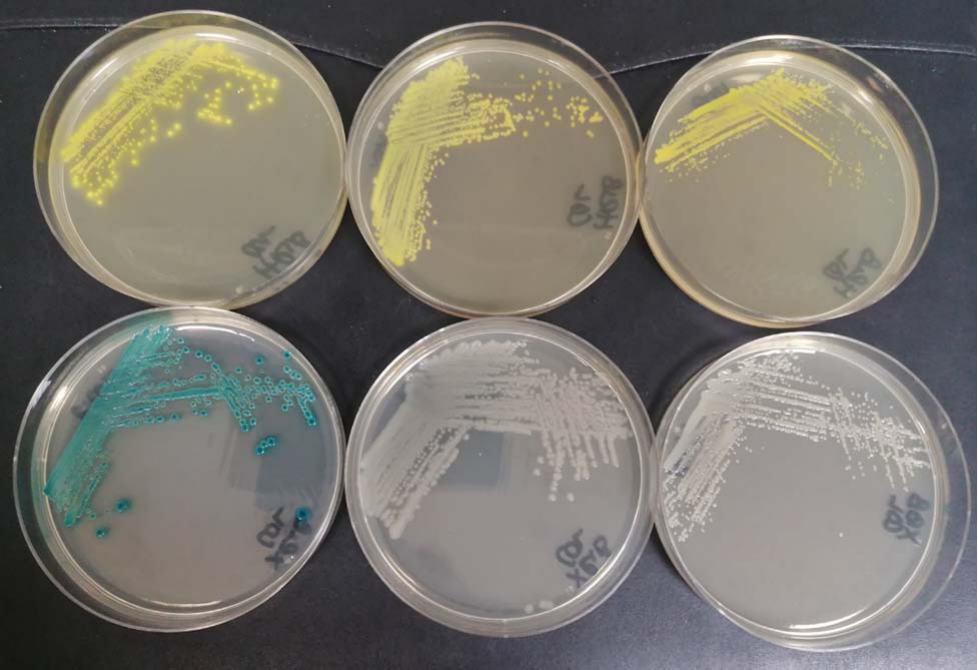
Substrate 22 (300 mg L^−1^) and X-riboside (80 mg L^−1^). Top plates, substrate 22; bottom plates, X-riboside. Left hand plates, *E. coli* (NCTC 10418); middle plates, *P. aeruginosa* (NCTC 12903); right hand plates, *B. cepacia* (NCTC 10743).

Each substrate was evaluated simultaneously against the 20 microorganisms listed in Table 1 as described previously.^8^ Each bacterial strain was inoculated onto Columbia blood agar and incubated at 37 °C overnight. *Candida* species were subcultured in the same way, but using Sabouraud agar. After incubation, a few colonies of each strain were suspended in sterile saline and the turbidity adjusted to 0.5 McFarland units using a Densimat (bioMérieux). 1 μL of each suspension was inoculated onto all of the test media using a multipoint inoculator that allowed the inoculation of 20 strains per plate. This resulted in an inoculum of approximately 100 000 colony-forming units (CFU) per spot. For selected strains, a 1 μL aliquot was inoculated onto a plate and spread over the full plate to obtain isolated colonies (see Fig. 3–5). Plates were incubated for 18 h at 37 °C and the appearance of visible growth and any colour produced were recorded. All tests were performed at least twice to ensure reproducibility.

**Table 1:** β-d-Galactosidase activity with substrates 10, 15 and 18

	Substrate	10		10		15		15		18		18	
	Colony colour	Black		Orange		Yellow		Yellow		Yellow		Yellow	
	Background colour	Pale yellow		Pale yellow		None		None		None		None	
	Ferric ammonium citrate (FAC) conc. (mg L^−1^)	500		0		500		0		500		0	
	Colony growth (G)^a^ and colour intensity (CI)^b^	G	CI	G	CI	G	Cl	G	CI	G	CI	G	CI
**Microorganism/reference** c													
*Gram-negative microorganisms*													
1	*Escherichia coli*	+	+	+	+	+	+	+	+	+	+	+	+
	NCTC 10418												
2	*Raoultella planticola*	+	+	+	+	+	+	+	+	±	±	+	+
	NCTC 9528												
3	*Providencia rettgeri*	+	−	+	−	+	−	+	−	+	−	+	−
	NCTC 7475												
4	*Enterobacter cloacae*	+	+	+	+	+	+	+	+	+	+	+	+
	NCTC 11936												
5	*Serratia marcescens*	+	Tr.	+	Tr.	+	±^e^	+	±^e^	+	+	+	+
	NCTC 10211												
6	*Salmonella typhimurium*	+	−	+	−	+	−	+	−	+	−	+	−
	NCTC 74												
7	*Pseudomonas aeruginosa*	+	−	+	−	+	−	+	−	+	−	+	−
	NCTC 10662												
8	*Yersinia enterocolitica*	+	−	+	−	+	−	+	−	+	+	+	+
	NCTC 11176												
9	*Burkholderia cepacia*	+	−	+	−	+	−	+	−	+	−	+	−
	NCTC 10743												
10	*Acinetobacter baumannii*	+	−	+	−	+	−	+	−	+	−	+	−
	NCTC 12156												
*Gram-positive microorganisms*													
11	*Streptococcus pyogenes*	+	−	+	−	+	−	+	−	−	−	−	−
	NCTC 8306												
12	*Staphylococcus aureus* (MRSA)	+	−	+	−	+	−	+	−	+	−	+	−
	NCTC 11939												
13	*Staphylococcus aureus* (MSSA)	+	−	+	−	+	−	+	−	+	−	+	−
	NCTC 6571												
14	*Staphylococcus epidermidis*	+	−	+	−	+	−	+	−	±	−	±	−
	NCTC 11047												
15	*Listeria monocytogenes*	+	−	+	−	+	−	+	−	+	−	+	−
	NCTC 11994												
16	*Enterococcus faecium*	+	−	+	−	+	−	+	−	+	+	+	+
	NCTC 7171												
17	*Enterococcus faecalis*	+	−	+	−	+	−	+	−	±	−	±	−
	NCTC 775												
18	*Bacillus atrophaeus* d	+	−	+	−	±	−	±	−	−	−	−	−
	ATCC 9372												
*Yeasts*													
19	*Candida albicans*	+	−	+	−	+	−	+	−	+	−	+	−
	ATCC 90028												
20	*Candida glabrata*	+	−	+	−	+	−	+	−	+	−	+	−
	NCPF 3943												

a + good growth, ± weak growth, Tr. trace of growth, − no growth. Growth on control plate was + (Gram-negative species) and + (Gram-positive species and yeasts).

b + strong colour, ± weak colour, Tr. trace of colour, − no colour.

c NCTC: National Collection of Type Cultures; ATCC: American Type Culture Collection; NCPF: National Collection of Pathogenic Fungi.

d Formerly named *Bacillus subtilis*.

e Orange.

Based on the results, extended testing was performed with 1-hydroxyanthraquinone β-d-galactopyranoside and 1-hydroxyanthraquinone β-d-glucopyranuronide. Both of these substrates were tested against a collection of *E. coli* isolates previously recovered from stool samples as well as an extended panel of Enterobacterales species (as documented in Tables S1 and S2†). In these extended tests, the substrates were tested at both 300 mg L^−1^ and 100 mg L^−1^. Equivalent substrates based on ‘X’ (5-bromo-4-chloro-indoxyl) were used as comparators and these were prepared as described below. This extended collection of strains was inoculated onto relevant plates exactly as described above. Agar plates containing X-β-ribofuranoside were also prepared so that the performance of 1-hydroxyanthraquinone β-d-ribofuranoside could be compared.

5-Bromo-4-chloro-3-indolyl-β-d-glucopyranuronide sodium salt (X-glucuronide), 5-bromo-4-chloro-3-indolyl-β-d-galactopyranoside (X-Gal) and 5-bromo-4-chloro-3-indolyl-β-d-ribofuranoside (X-riboside) were all obtained from Glycosynth, Warrington, UK and tested alongside equivalent substrates based on 1-hydroxyanthraquinone. They were incorporated into Columbia agar exactly as described above but without the inclusion of FAC, and using 8 mg of substrate instead of 30 mg, thus giving a final concentration of 80 mg L^−1^.

## Results and discussion

Table 1 indicates good microbial growth on Columbia agar in the presence of all the β-galactosidase substrates apart from *Streptococcus pyogenes* and *Bacillus atrophaeus* whose growth was inhibited by substrate 18 in both the presence and absence of FAC. Three other microorganisms (*Raoultella planticola*, *Staphylococcus epidermidis* and *Enterococcus faecalis*) only displayed weak growth with substrate 18 and *Bacillus atrophaeus* was the only microorganism which grew weakly in the presence of substrate 15.

In the presence of quinizarin di-β-d-galactopyranoside 10, FAC, and IPTG three *Enterobacterales* (*Escherichia coli*, *Raoultella planticola* and *Enterobacter cloacae*) produced well-defined, black colonies which we suggest is due to chelation of the aglycone 6 with ferric ions (Table 1). Only a trace of colour was observed for *Serratia marcescens* and none of the other microorganisms listed in Table 1 produced any discernible colouration. The nature of the iron species did not appear to be important; black chelates were also observed using iron(ii) acetylacetonate, iron(iii) acetylacetonate and iron(ii) acetate as illustrated with *E. coli* (Fig. 3). When FAC was omitted from the medium, the same four microorganisms noted above produced orange colonies with comparable colour intensities (Table 1). The orange colour was attributed to the aglycone 6. This is exemplified in the top-right image of Fig. 3 for *E. coli*. When both FAC and IPTG were absent from the medium, good colony colouration was also observed suggesting the presence of IPTG is not critical (Fig. 3, bottom right image).

The anthrarufin di-β-d-galactopyranoside 15 enabled the detection of *Escherichia coli*, *Raoultella planticola* and *Enterobacter cloacae* as did the quinizarin substrate 10. Rather unexpectedly, black chelates were not observed when FAC was incorporated in the medium and strongly coloured, yellow colonies were apparent. Similar results were also obtained in the absence of FAC, suggesting that the colour could be attributed to the presence of the aglycone 11 alone. *Serratia marcescens* was also detected by substrate 15 producing weakly, yellow-coloured colonies.

**Scheme 1:**
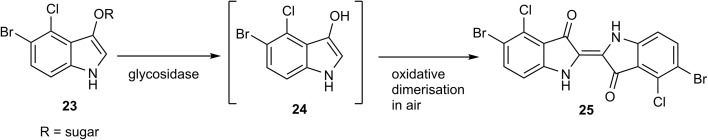
The ‘X’-series of indoxylic glycosidase substrates.

Similarly to substrate 15, 1-hydroxyanthraquinone β-d-galactopyranoside 18 also afforded yellow microbial colonies in both the presence and absence of FAC when galactosidase activity was present. *Escherichia coli*, *Raoultella planticola* and *Enterobacter cloacae* resulted in strong yellow colonies in the absence of FAC whereas in the presence of FAC, *Raoultella planticola* produced only weakly coloured colonies. In contrast to the substrates 10 and 15, *Yersinia enterocolitica* and *Enterococcus faecium* produced coloured colonies with substrate 18. A comparative study of this substrate against X-Gal (5-bromo-4-chloro-3-indolyl-β-d-galactopyranoside 23) (Scheme 1) using 40 different strains of *E. coli* was conducted and 12 additional strains of Enterobacterales (see ESI, Tables S1 and S2†). X-Gal is commonly employed in molecular biology^13^ and microbial diagnostics^14^ to test for the presence of the β-d-galactosidase enzyme. After hydrolysis of the substrate, the resulting indoxylic aglycone 24 undergoes oxidative dimerization in air to form the coloured dye 25. With *E. coli*, the performance of substrate 18 (300 mg L^−1^) and X-Gal (80 mg L^−1^) were judged to be comparable producing yellow and green-coloured colonies respectively. Substrate 18 was also effective at a concentration of 100 mg L^−1^ although the colonies' colour intensity was diminished. Fig. 4 depicts *E. coli* (NCTC 12241) in the presence of substrate 18 and X-Gal. For the 12 additional strains of *Enterobacterales*, identical results were produced for substrate 18 and X-Gal with the exception that substrate 18 caused inhibition of the growth of *Hafnia alvei* (Table S2†).

Based on these results, 1-hydroxyanthraquinone β-d-galactopyranoside was considered to be an effective substrate for detection of β-d-galactosidase in *E. coli* and other species of Enterobacterales (also known as “coliforms”). Substrates targeting β-d-galactosidase are highly useful for detection of coliforms in a wide range of sample types including clinical samples,^15^ water samples,^16^ and food.^17^ Such substrates are frequently combined with one or more other chromogenic substrates^18^ to provide differentiation of different bacterial species and it is usually advantageous to utilise substrates based on different coloured chromophores. There are very few chromogenic substrates suitable for use in agar that result in the formation of yellow colonies. A limitation of chromogenic substrates based on indoxyl compounds such as ‘X’ is that coloured colonies will not be produced under anaerobic conditions due to the requirement for oxygen to produce coloration. This is not a limitation for substrates based on 1-hydroxyanthraquinone and there is interest in clinical microbiology in the detection of anaerobic bacteria that do not grow in the presence of oxygen.^19,20^

Glucopyranuronide substrates are commonly used in diagnostic microbiology to detect the presence of *E. coli* because most strains of this species produce β-glucuronidase, which distinguishes them from the vast majority of other Gram-negative bacteria.^2^ In view of the results noted above using the β-d-galactopyranoside substrate 18 for *E. coli* detection, a similar study was therefore initiated using the β-d-glucopyranuronide substrate 20 and the same 40 strains of *E. coli* employed in the aforementioned study (see ESI, Table S3†). Substrate 20 performed considerably better at a higher concentration (300 mg L^−1^*versus* 100 mg L^−1^) with five more *E. coli* strains (23 in total) being detected. When compared with the sodium salt of X-glucuronide (5-bromo-4-chloro-3-indolyl-β-d-glucopyranuronide) which enabled the detection of 33 *E. coli* strains at a concentration of 80 mg L^−1^, substrate 20 was deemed to be significantly less effective.

We have previously shown that β-ribosidase activity is widely spread across many genera of Gram-negative bacteria.^21^ Within this study, some differences in β-d-ribosidase activity were observed that were dependent on the nature of the substrates' aglycone; *e.g.* 97% of 74 *Pseudomonas aeruginosa* strains were detected when using 3′,4′-dihydroxyflavone-4-β-d-ribofuranoside (DHF-β-riboside) whereas 5-bromo-4-chloro-3-indolyl-β-d-ribofuranoside (X-riboside) was ineffective in detecting β-ribosidase activity in any of these strains. It is therefore of value to continue to design enzyme substrates with a variety of aglycones as this can significantly affect their utility. Hence, it was of interest to compare the performances of substrate 22 and X-riboside against a range of microorganisms. 12 Gram-negative and 8 Gram positive microorganisms were therefore selected for evaluation against these two substrates (Table 2). The growth of all microorganisms was good in the presence of X-riboside. Most of the Gram-negative microorganisms exhibited good growth in the presence of substrate 22 apart from *Stenotrophomonas maltophilia* whose growth was inhibited and *Vibrio parahaemolyticus* whose growth was poor. With the exception of *Enterococcus faecium*, the growth of the Gram-positive microorganisms was generally inhibited or weak. In the presence of X-riboside, six of the Gram-negative microorganisms were associated with the production of strongly coloured, blue/green colonies. These microorganisms also resulted in the formation of strongly coloured, yellow colonies in the presence of substrate 22. *Pseudomonas aeruginosa* and *Burkholderia cepacia* also produced coloured colonies with substrate 22 but not with X-riboside (Fig. 5). Although Table 2 suggests that substrate 22 may be useful for the detection of *Burkholderia cepacia*, out of 34 strains examined in the previous study,^21^ only 53% gave coloured colonies with the most sensitive substrate tested, DHF-β-riboside. This casts doubt on the potential of β-ribosidase activity to serve as a useful diagnostic marker for this species.

**Table 2:** Detection of β-d-ribosidase activity using substrate 22 and X-riboside. Incubation time 22 h

	Substrate	22		X-Riboside	
	Colony colour	Yellow		Blue/green	
	Background colour	Pale yellow		None	
	Substrate conc. (mg L^−1^)	300		80	
	Colony growth (G)^a^ and colour intensity (CI)^b^	G	CI	G	CI
**Microorganism/reference** c					
*Gram-negative microorganisms*					
1	*Escherichia coli*	+	+	+	+
	NCTC 10418				
2	*Raoultella planticola*	+	+	+	+
	NCTC 9528				
3	*Providencia rettgeri*	+	+	+	+
	NCTC 7475				
4	*Enterobacter cloacae*	+	+	+	+
	NCTC 11936				
5	*Serratia marcescens*	+	+	+	+
	NCTC 10211				
6	*Salmonella enteritidis*	+	+	+	+
	NCTC 6676				
7	*Pseudomonas aeruginosa*	+	+	+	−
	NCTC 12903				
8	*Yersinia enterocolitica*	+	−	+	−
	NCTC 11176				
9	*Burkholderia cepacia*	+	+	+	−
	NCTC 10743				
10	*Acinetobacter baumannii*	+	Tr.	+	−
	NCTC 12156				
11	*Stenotrophomonas maltophilia*	−	−	+	−
	NCTC 10257				
12	*Vibro parahaemolyticus*	Tr.	−	+	−
	NCTC 10903				
*Gram-positive microorganisms*					
13	*Streptococcus pyogenes*	−	−	+	−
	NCTC 8306				
14	*Staphylococcus aureus* (MRSA)	−	−	+	±
	NCTC 11939				
15	*Staphylococcus aureus* (MSSA)	−	−	+	+
	NCTC 6571				
16	*Staphylococcus epidermidis*	Tr.	−	+	−
	NCTC 11047				
17	*Listeria monocytogenes*	±	−	+	−
	NCTC 11994				
18	*Enterococcus faecium*	+	−	+	−
	NCTC 7171				
19	*Enterococcus faecalis*	±	−	+	−
	NCTC 12697				
20	*Bacillus atrophaeus* d	−	−	+	−
	ATCC 9372				

a + good growth, ± weak growth, Tr. trace of growth, − no growth. Growth on control plate was + (Gram-negative species) and + (Gram-positive species).

b + strong colour, ± weak colour, Tr. trace of colour, − no colour.

c NCTC: National Collection of Type Cultures; ATCC: American Type Culture Collection.

d Formerly named *Bacillus subtilis*.

When using a cocktail of complementary chromogenic substrates in solid (agar) media, it is important to also be able to select from a range of complementary colours that colonies of different species are able to produce. Chromogenic glycosidase substrates that yield a non-diffusible yellow or orange colour are relatively uncommon with only a few examples available including the Aldol® enzyme substrates.^4,22^ The 1-hydroxyanthraquinone-based substrates described above would therefore have potential in this area. One potential disadvantage of the 1-hydroxyanthraquinone substrates is that a relatively high concentration of substrate (up to 300 mg L^−1^) is required to obtain a strong coloration, which has economic implications, and there is some evidence of growth inhibition of certain species; such as *Staphylococcus aureus* and *Streptococcus pyogenes* being inhibited by substrate 22 at 300 mg L^−1^ (see Table 2). This would likely limit the application of 1-hydroxyanthraquinone-based substrates in chromogenic culture media that are required to grow a wide range of species, but could prove advantageous for a culture medium that targets a particular species for which growth is unaffected. Further work could establish the optimal concentration of 1-hydroxyanthraquinone-based substrates that would allow growth and coloration (where applicable) for a range of individual species.

## Conclusion

The substrates described in this paper all resulted in coloured microbial colonies (when appropriate microbial enzyme activity was present) with excellent colour contrast to the background. The colour generated by microbial colonies was localised and did not diffuse in the agar medium. In the absence of iron salts, all the substrates examined produced orange or yellow-coloured microbial colonies attributed to the presence of the corresponding aglycone. When iron salts were present in the medium, rather unexpectedly, only the quinizarin di-β-d-galactopyranoside 10 appeared to produce a chelated aglycone, and the mechanism of these colour changes may be further investigated. In extended studies on *E. coli* detection, 1-hydroxyanthraquinone-β-d-galactopyranoside 18 performed well against X-Gal but its glucuronide derivative 20 was less effective than X-glucuronide. Its ribose derivative 22 does however show potential for *Pseudomonas aeruginosa* detection when compared to X-riboside. With relatively few commercially available glycoside substrates with yellow endpoints, some of the compounds described in this paper could find use when two or more chromogenic substrates producing complementary colours are required.

## Data availability

The data supporting this article have been included as part of the ESI.†

## Author contributions

Conceptualisation; MB, JDP, synthetic work and data analysis; AG, HJT, SPS, GT, microbiological work and data analysis, AG, ECLM, JDP, HJT; project management MB, JDP, SPS, GT, HJT, writing manuscript MB, JDP, SPS, GT.

## Conflicts of interest

This study was funded by Glycosynth Ltd and Northumbria University.

## Supplementary Material

RA-015-D4RA06418F-s001
