# Supplementary material for: Chromogenic hydroxyanthraquinone-based enzyme substrates for the detection of microbial β-d-galactosidase, β-d-glucuronidase and β-d-ribosidase
Source: RSC Adv. 2025 Feb 7;15(6):4229–35. doi: 10.1039/d4ra06418f (PMC11804412; doi:10.1039/d4ra06418f)
Supplement: RA-015-D4RA06418F-s001 [file RA-015-D4RA06418F-s001.pdf]

Supplementary Information (SI) for RSC Advances.
This journal is © The Royal Society of Chemistry 2025

Chromogenic hydroxyanthraquinone-based enzyme substrates for the detection of microbial β-D-galactosidase, β-D-glucuronidase and β-D-ribosidase

Michael Burton, Amy Garcha, Emma C. L. Marrs, John D. Perry, Stephen P. Stanforth, Graeme Turnbull and Hayley J. Turner

Supplementary information

NMR spectra were recorded on a Jeol spectrometer at either 270 or 400 MHz for ^{1}H spectra and either 68 or 100 MHz for ^{13}C spectra. All chemical shifts are quoted in ppm relative to tetramethylsilane. Optical rotations were measured on an Optical Activity AA10 polarimeter. High resolution mass spectra (HRMS) were obtained from the EPSRC Mass Spectrometry Facility (Swansea). Flash chromatography was performed using Fluorochem Ltd silica gel (60 Å). Mixed solvents are recorded as volumetric ratios.

Compounds **7** and **9** have been reported previously in the literature; X. Sun, L. Ji, S. Ren, S. Wan and T. Jiang, *Synth. Commun*., 2008, **38**, 4116-4124 (ref. 11 in text).

Quinizarin-2′,3′,4′,6′-tetra-*O*-acetyl-β-D-galactopyranoside **7** and quinizarin-di-(2′,3′,4′,6′-tetra-*O*-acetyl-β-D-galactopyranoside) **9**.

A mixture of quinizarin **6** (5 g, 20.8 mmol), acetobromo-α-D-galactopyranose (AB-Gal) (18.95 g, 46 mmol) and tetra-*n*-butyl ammonium bromide (TBAB) (14.67 g, 45.5 mmol) was stirred in CH_2Cl_2 (350 mL). An aqueous solution of NaOH (1.75 g, 43.7 mmol) in deionised water (DI) (150 mL) was added dropwise at RT over approximately 3 hours. Initially, the two-phase reaction mixture was purple in colour and the pH of the aqueous layer was >10. After stirring at RT for 4 h, the organic layer had formed a clear orange solution and the aqueous layer was clear and colourless and the pH of the aqueous layer had dropped to ~6. The reaction mixture was stirred overnight at RT. TLC of the reaction mixture showed it contained two possible product spots as well as a large amount of unreacted quinizarin. Further portions of AB-Gal (10 g, 24.3 mmol) and TBAB (14 g, 43.4 mmol) were added followed by an aqueous solution of NaOH (1.75 g, 43.7 mmol) in DI water (50 mL). The reaction mixture was stirred overnight at RT and the reaction mixture was poured into CH_2Cl_2 (200 mL). The organic portion was washed sequentially with 1 M aqueous NaOH solution (6 x 500 mL) and DI water (3 x 500 mL) before being dried ($MgSO_4$) and concentrated under vacuum to give a red/brown solid (12.01 g). The resulting solid was triturated in acetone yielding a yellow solid with R_f 0.45 (5.36 g). The filtrate was concentrated giving a mixture of products with R_f 0.45 and R_f 0.25 (using toluene/acetone 5:1 as the eluent) as a black oil (5.33 g).

The yellow solid (5.36 g, 9.4 mmol), AB-Gal (6 g, 14.6 mmol) and TBAB (4.50 g, 14 mmol) were stirred in CH_2Cl_2 (200 mL). NaOH (0.57 g, 14.2 mmol) in DI water (70 mL) was added in one portion and the reaction mixture was stirred overnight at RT. TLC showed that although the majority of the high running product (R_f 0.45) had been converted to the low running product (R_f 0.25), the reaction mixture still contained unreacted AB-Gal. The pH of the aqueous NaOH layer had dropped to ~6. NaOH (0.60 g, 15 mmol) in DI water (25 mL) was then added in one portion and the mixture was

stirred at RT overnight. The reaction mixture was poured into CH_2Cl_2 (150 mL) and the organic portion was washed with DI water (3 x 400 mL), dried ($MgSO_4$) and concentrated producing a yellow semi-solid. This semi-solid was combined with the previously obtained black oil and purified by flash chromatography using C_{60} silica gel (500 g). The column was eluted with toluene/acetone 5:1 (~6 L) collecting fractions of ~150 mL. Fractions 5-11 were combined and concentrated yielding an orange solid (1.58 g). This solid was triturated with acetone and the resulting yellow powder collected yielding compound **7** (540 mg, 4.5%), m.p. 257-258 °C [lit. 106-110 °C]. $[\alpha]_D^{30}$ –88° (*c* 1 in $CHCl_3$). ^{1}H-NMR: (DMSO-d_6) δ 8.27 and 8.20 (1H, dd, *J*=2.3 Hz, *J*=6.4 Hz, Ar-H5/8), 7.78 (2H, m, Ar-H6/7), 7.61 and 7.44 (1H, d, *J*=9.2 Hz, Ar-H2/3), 5.63 (1H, dd, *J*=10.5 Hz, *J*=8.2 Hz, H-2), 5.45 (1H, d, *J*=3.7 Hz, H-4), 5.31 (1H, dd, *J*=10.5 Hz, *J*=3.2 Hz, H-3), 5.02 (1H, d, *J*=7.8 Hz, H-1), 4.23 (1H, dd, *J*=11.2 Hz, *J*=7.1 Hz, H-6a), 4.13 (1H, dd, *J*=11.4 Hz, *J*=6.4 Hz, H-6b), 3.97 (1H, m, H-5), 2.23, 2.17, 2.03, 2.02 (12H, 3 x s, CH_3C=O). Although there is a large difference in the melting point reported by Sun *et al.* the ^{1}H-NMR spectrum of the product was consistent with that reported by these authors.

Fractions 16-30 were combined and concentrated producing an orange-coloured, foaming solid (7.14 g). The foam was dissolved in boiling IMS (50 mL) and the orange solution kept at 4 °C overnight. The resulting orange powder was collected (5.52 g) and was dissolved in boiling IMS (75 mL). Charcoal (1 g) was added, and the mixture filtered through pre-washed Celite. The resulting solution was allowed to cool to RT and the yellow powder collected by filtration to give the compound **9** (3.57 g, 19%), m.p. 142-144 °C [lit. 214-217 °C]. $[\alpha]_D^{30}$ –50° (*c* 0.5 in $CHCl_3$). ^{1}H-NMR: (DMSO-d_6) δ 8.09 (2H, dd, *J*=6.0 Hz, *J* 3.7 Hz, Ar-H5/8), 7.71 (2H, dd, *J*=5.5 Hz, *J*=3.2 Hz, Ar-H6/7), 7.53 (2H, s, Ar-H2/3), 5.65 (2H, dd, *J*=10.5 Hz, *J*=7.8 Hz, H-2), 5.47 (2H, d, *J*=2.70 Hz H-4), 5.13 (2H, dd, *J*=10.2 Hz, *J*=3.2 Hz, H-3), 5.06 (2H, d, *J*=8.2 Hz, H-1), 4.25 (2H, dd, *J*=11.2 Hz, *J*=6.6 Hz, H-6a), 4.13 (2H, dd, *J*=11.2 Hz, *J*=6.6 Hz, H-6b), 4.01 (2H, m, H-5), 2.20, 2.18, 2.03, 2.02 (24H, 4 x s, CH_3C=O). As with the mono-galactoside **7** there is a large difference in the melting point reported by Sun *et al.* but the ^{1}H-NMR spectrum was consistent with that reported by these authors.

Quinizarin di-β-D-galactopyranoside **10.**

Compound **9** (1.5 g, 1.6 mmol) was suspended in MeOH (7 mL) and a NaOMe solution in methanol (2.17 M, 0.03 mL) was added. The mixture changed from pale yellow to deep red and was kept overnight at 4 °C. TLC showed complete deacetylation. The red mixture was neutralised with AcOH (0.1 mL). The resulting yellow solid was collected giving the desired product compound **10** (910 mg, 96.8%), m.p. 145-146 °C. $[\alpha]_D^{30}$ –42° (*c* 0.5 in DMSO). ^{1}H-NMR: (DMSO-d_6) δ 8.03 (2H, dd, *J*=5.2 Hz, *J*=3.5 Hz, Ar-H5/8), 7.84 (2H, dd, *J*=5.4 Hz, *J*=3.2 Hz, Ar-H6/7), 7.69 (2H, s, Ar-H2/3), 5.01 (2H, d, *J*=3.0 Hz, OH), 4.99 (2H, d, *J*=6.0 Hz, OH), 4.96 (2H, d, *J*=7.7 Hz, H-1), 4.72 (2H, t, *J*=5.3 Hz, OH), 4.63 (2H, d, *J*=4.0 Hz, OH), 3.71 (4H, m, H-2/4), 3.65 (2H, m, H-3), 3.57 (4H, m, H-6a/6b), 3.43 (2H, m, H-5). ^{13}C NMR (DMSO-d_6): δ 183.0 (C=O quinizarin), 153.0, 134.4, 126.6, 125.4, 123.0 (quinizarin), 103.1 (C-1), 76.5, 73.6, 71.0, 68.7, 61.0 (C-2/3/4/5/6). HRMS (ESI) for $C_{26}H_{28}O_{14}Na$ $[M+Na]^+$: *m/z* calcd 587.1317; measured: 587.1366.

Anthrarufin 2′,3′,4′,6′-tetra-*O*-acetyl-β-D-galactopyranoside **12** and anthrarufin di-(2,3,4,6-tetra-*O*-acetyl-β-D-galactopyranoside) **14**.

A mixture of anthrarufin **11** (5 g, 20.8 mmol), AB-Gal (18 g, 43.8 mmol) and TBAB (14 g, 43.5 mmol) was stirred in CH_2Cl_2 (350 mL). NaOH (1.79 g, 44.8 mmol) in DI water (150 mL) was then added dropwise over 1 h. The two-phase reaction was stirred overnight at RT. A TLC of the organic layer showed the reaction mixture contained a small amount of possible product and a large amount of unreacted anthrarufin and AB-Gal. A further portion of NaOH (1.75 g, 44.8 mmol) in DI water (150 mL) was then added dropwise over 1 h. The mixture was stirred for 2 h at RT. A TLC of the organic layer looked unchanged. TBAB (7 g, 21.7 mmol) was added, and the reaction mixture stirred for 1 h at RT. Further NaOH (1.75g, 44.8 mmol) in DI water (50 mL) was added dropwise over 1 h and the mixture was stirred overnight at RT. The reaction mixture was poured into a mixture of aqueous sat. $NaHCO_3$ (250 mL) and CH_2Cl_2 (150 mL). The organic layer was separated and washed sequentially with aqueous 1 M NaOH solution (3 x 300 mL) and DI water (2 x 500 mL) and was then dried ($MgSO_4$) and concentrated to produce a brown oil (11.42 g). This oil was purified by flash chromatography using C_{60} silica gel (500 g). The column was eluted with toluene/acetone (5:1) (~5.5 L), followed by toluene/acetone (1:1) (~3 L) collecting fractions of ~150 mL. Fractions 8-24 were combined and concentrated yielding a yellow solid (3.79 g). This solid was triturated with MeOH and the resulting solid was purified by recrystallisation from MeOH/DI water (150 mL/50 mL) using charcoal (500 mg). The resulting yellow powder was collected giving compound **12** (1.96 g, 16.5%). m.p. 205-206 °C. $[\alpha]_D^{24}$ -80° (*c* 0.1 in $CHCl_3$). ^{1}H-NMR: (DMSO-d_6) δ 12.24 (1H, s, OH), 7.98 (1H, d, *J*=6.4 Hz, Ar-H8), 7.91 (1H, t, *J*=7.9 Hz, Ar-H4), 7.78 (1H, t, *J*=7.7 Hz, Ar-H7), 7.67 (1H, d, *J*=8.4 Hz, Ar-H3), 7.60 (1H, d, *J*=7.7 Hz, Ar-H6), 7.32 (1H, d, *J*=8.2 Hz, Ar-H2), 5.63 (1H, d, *J*=7.9 Hz, H1), 5.38 (2H, m, *J*=10.4 Hz, *J*=7.7 Hz, H2/4), 5.29 (1H, dd, *J*=10.4 Hz, *J*=3.2 Hz, H3), 4.50 (1H, t, *J*=6.2 Hz, H5), 4.10 (2H, m, H-6a/6b), 2.16, 2.03, 2.02, 1.95 (4 x 1H, 4 x s, C*H*$_3$C=O). ^{13}C NMR: (DMSO-d_6) δ 188.3, 180.7 (C=O anthrarufin), 170.5, 170.4, 170.1, 169.5 (CH_3*C*=O), 161.5 157.1, 137.9, 135.8, 135.3, 135.2, 124.1, 123.3, 122.5, 121.9, 119.2, 115.9 (anthrarufin), 98.8 (C-1), 71.2, 70.8, 68.4, 67.8, 61.9 (C-2/3/4/5/6), 21.1, 21.0, 21.0, 20.9 (*C*H$_3$C=O). HRMS (ESI) for $C_{28}H_{26}O_{13}N$ $[M+NH_4]^+$: *m/z* calcd 588.1712; measured: 588.1717.

Fractions 27 to 49 were combined and concentrated under vacuum to an orange foaming solid (4.38 g). This solid was purified by flash chromatography using C_{60} silica gel (200 g). The column was eluted with toluene/acetone (5:1) (~ 5.5 L) collecting fractions of ~ 50 mL. Fractions 21-41 were combined and concentrated giving an orange foam (3.24 g). This foam was triturated with MeOH (20 mL) and the resulting pale-yellow solid was collected yielding compound **14** (2.15 g, 11.5%), m.p. 153-155 °C. $[\alpha]_D^{28}$ -70° (*c* 0.1 in MeOH). ^{1}H-NMR: (DMSO-d_6) δ 7.90-7.78 (4H, m, Ar-H), 7.57 (2H, dd, *J*=8.2, *J*=3.9 Hz, Ar-H), 5.60 (2H, d, *J*=7.7 Hz, H-1), 5.39 (4H, m, *J*=10.1 Hz, H-2/4), 5.29 (2H, dd, *J*=10.4 Hz, *J*=3.2 Hz H-3), 4.5 (2H, t, *J*= 6.3 Hz, H-5) 4.13 (4H, m, *J*=6.3 Hz, H-6a/6b), 2.16, 2.04, 2.01, 1.95 (4 x 6H, 4 x s, C*H*$_3$C=O). ^{13}C NMR: (DMSO-d_6) δ 181.2 (*C*=O anthrarufin), 170.5, 170.4, 170.1, 169.5 (CH_3*C*=O) 156.4, 137.1, 135.7, 122.4, 121.9, 121.7 (anthrarufin), 98.8 (C-1), 71.2, 70.8, 68.7, 67.6, 61.9 (C-2/3/4/5/6), 21.2, 21.0, 21.0, 20.9 (*C*H$_3$C=O). HRMS (ESI) for $C_{42}H_{48}O_{22}N$ $[M+NH_4]^+$: *m/z* calcd 918.2662; measured: 918.2665.

Anthrarufin di-β-D-galactopyranoside **15.**

Compound **14** (1.5 g, 1.6 mmol) was suspended in MeOH (4.5 mL) and NaOMe solution in methanol (2.17 M, 0.5 mL) was added. The mixture was kept at RT overnight. After three hours the mixture had set completely solid and MeOH (10 mL) was added to keep the suspension mobile. A TLC showed complete deacetylation had occurred. The resulting yellow solid was collected and was added to a mixture of boiling MeOH (100 mL) and DI water (50 mL); the solid did not fully dissolve and additional quantities of MeOH (50 mL) and DI water (100 mL) were therefore added. The solution was boiled for 5 min. before being cooled in the fridge at 4 °C overnight. The resulting yellow, fluffy solid was collected by filtration yielding compound **15** (890 mg, 95%). m.p. 244-245 °C. $[\alpha]_D^{30}$ -49° (*c* 1 in $CHCl_3$). ^{1}H-NMR: (DMSO-d_6) δ 7.80 (4H, m, Ar-H-8/4/7/3), 7.63 (2H, d, *J*=7.2 Hz, Ar-H6/2), 5.01 (2H, m, H-2/4), 4.94 (1H, d, *J*=5.7 Hz, H-1), 4.70 (2H, t, *J*=5.4 Hz, H-3), 4.63 (1H, d, *J*=4.0 Hz, H-5), 3.80-3.43 (2H, m, H-6a/6b). ^{13}C NMR: (DMSO-d_6) δ 182.1 (*C*=O anthrarufin), 157.8, 137.1, 135.8, 122.0, 121.5, 120.8 (anthrarufin), 102.1 (C-1), 76.4, 73.4, 70.9, 68.6, 60.9 (C-2/3/4/5/6). HRMS (ESI) for $C_{26}H_{28}O_{14}Na$ $[M+Na]^+$: *m/z* calcd 587.1371; measured: 587.1368.

1-Hydroxyanthraquinone 2′,3′,4′,6′-tetra-*O*-acetyl-β-D-galactopyranoside **17**.

A mixture of 1-hydroxyanthraquinone **16** (4.66 g, 20.8 mmol), AB-Gal (9.39 g, 22.8 mmol) and TBAB (7.36 g, 22.8 mmol) was stirred in CH_2Cl_2 (175 mL). An aqueous solution of NaOH (914 mg, 22.8 mmol) in DI water (80 mL) was then added. The two-phase, deep, red-coloured reaction mixture was stirred at RT for 6 h. A TLC of the reaction mixture indicated product formation. The CH_2Cl_2 layer was separated, washed sequentially with aqueous 1 M NaOH (6 x 500 mL) and DI water (2 x 500 mL), and then dried ($MgSO_4$) and concentrated yielding a yellow solid (16.66 g). This solid was triturated with acetone (50 mL) and the resulting yellow powder was collected and then washed with a little acetone. The yellow solid was purified by recrystallisation from boiling acetone (175 mL) giving compound **17** as a pale-yellow solid (3.47 g, 30%). m.p. 241-243 °C. $[\alpha]_D^{24}$ -58° (*c* 1 in acetone). ^{1}H-NMR: (DMSO-d_6) δ 8.07 (2H, dd, *J*=7.3 Hz, *J*=1.9 Hz, Ar-H5/8), 7.86 (4H, m, Ar-H6/7/3/4), 7.63 (1H, dd, *J*=8.2 Hz, *J*=0.9 Hz, Ar-H2), 5.59 (1H, d, *J*=7.8 Hz, H1), 5.37 (2H, m, H2/4), 5.25 (1H, dd, *J*=10.5 Hz, *J*=3.2 Hz, H-3), 4.46 (1H, t, *J*=6.4 Hz, H5), 4.10 (2H, m, H-6a/6b), 2.13, 2.01, 1.99, 1.92 (4 x 1H, 4 x s, C*H*$_3$C=O). ^{13}C NMR: (DMSO-d_6) δ 189.0, 181.0 (*C*=O 1-hydroxyanthraquinone), 170.6, 170.1, 169.9, 169.6 (CH$_3$*C*=O), 158.5, 150.3, 135.8, 134.6, 134.4, 132.3, 129.8, 127.2, 126.6, 126.1, 124.3, 116.1 (1-hydroxyanthraquinone), 99.2 (C-1), 72.5, 71.5, 70.9, 68.5, 62.2 (C2/3/4/5), 21.1, 21.0, 20.9, 20.9 (*C*H$_3$C=O). HRMS (ESI) for $C_{28}H_{30}O_{12}N$ $[M+NH_4]^+$: *m/z* calcd 572.1763; measured: 572.1761.

1-Hydroxyanthraquinone β-D-galactopyranoside **18**.

Compound **17** (2.5 g, 4.5 mmol) was suspended in MeOH (6 mL) and a NaOMe solution in methanol (2.17 M, 1 mL) was added. After ~10 min. the reaction mixture had set completely solid, therefore methanol (15 mL) was added and the mixture kept at RT for 48 h, at which point a TLC showed complete deprotection had occurred. The reaction mixture was neutralised to pH ~5 using AcOH (0.5 mL) and the resulting solid was collected and washed with MeOH (0.5 mL) affording compound **18** as a yellow powder (1.46 g, 84%). m.p. 212.5-215.5 °C. $[\alpha]_D^{24}$ -28° (*c* 1 in DMSO). ^{1}H-NMR: (DMSO-d_6) δ 8.07 (2H, t, *J*=6.6 Hz, Ar-H5/8), 7.83 (4H, m, Ar-H-3/4/6/7), 7.68 (1H, dd, *J*=7.3 Hz, *J*=0.9 Hz, Ar-H2),

5.05 (2H, d, *J*=7.8 Hz, H-1/OH), 4.78 (2H, broad s, OH), 3.73 (2H, m, H-2/4), 3.65 (1H, t, *J*=6.4 Hz, H-5), 3.52 (2H, m, H-6a/6b), 3.44 (1H, dd, *J*=9.6 Hz, *J*=3.2 Hz, H-3). HRMS (ESI) for $C_{20}H_{18}O_8Na$ $[M+Na]^+$: *m/z* calcd 409.0894; measured: 409.0895.

1-Hydroxyanthraquinone 2′,3′,4′-tri-O-acetyl-β-D-glucopyranuronide-6′-methyl ester **19**.

A mixture of 1-hydroxyanthraquinone **16** (31.95 g, 142.5 mmol) and 3 Å molecular sieves (71.25 g) was stirred in DCM (570 mL). Collidine (175 mL, 1.32 mol) was then added, followed by Ag_2CO_3 (50 g, 181.3 mmol). The mixture was stirred for 10 minutes before acetobromo-α-D-glucopyranuronic acid methyl ester (ABU, 71.25 g, 179.4 mmol) was added. The mixture was stirred at RT in the dark for 48 h. TLC showed a substantial amount of unreacted starting material, and so additional Ag_2CO_3 (20 g) was added and the mixture stirred overnight in the dark. TLC showed little product formation, and so additional collidine (80 mL) was added and the mixture again stirred overnight in the dark. TLC then showed no remaining ABU and good product formation. The mixture was filtered through pre-washed celite, washed with 1 M HCl (2 x 2L), then sodium bicarbonate (4 x 2 L), then 1 M NaOH (2 x 2L), and DI water (2 x 2 L), before being dried over $MgSO_4$ and concentrated under reduced pressure to a brown/orange foaming oil which set solid overnight (414 g). The isolated solid was triturated in hot IMS (1.4 L), cooled to -20 °C overnight, and crude product collected by filtration (41.0 g). The obtained yellow solid was purified by recrystallisation twice from boiling IMS (1.7 L) and charcoal (15 g). The resulting product was filtered, washed with IMS, and dried in air to afford compound **19** as a yellow solid (25.75 g, 33%). m.p. 199.8-201.3 °C. Rotation. $[\alpha]_D^{20}$ - 102° (*c* 0.5 in acetone). ^{1}H-NMR: ($CDCl_3$) δ 8.23-8.19 (2H, m, 2 x Ar-H), 8.12 (1H, dd, *J*=7.6 Hz, *J*=1.1 Hz), 7.79-7.69 (3H, m, 3 x Ar-H), 7.61 (1H, dd, *J*=8.2 Hz, *J*=0.9 Hz), 5.50-5.46 (1H, m, 1 x H), 5.44-5.36 (2H, m, 2 x H), 5.25 (1H, d, *J*=7.3 Hz, 1 x H), 4.20 (1H, d, *J*=9.2 Hz, 1 x H), 3.72 (3H, s, CO_2CH_3), 2.17, 2.09, 2.04 (3 x 3H, 3 x s, CH_3C=O). ^{13}C NMR: ($CDCl_3$) δ 183.0, 181.5 (*C*=O 1-hydroxyanthraquinone), 170.2, 169.8, 169.5 (CH_3C=O), 167.0 (C-6), 156.5, 135.5, 134.8, 134.7, 134.4, 133.6, 132.5, 127.3, 126.8, 125.7, 123.7, 123.4, 100.1 (C-1), 72.7, 71.7, 70.8, 69.1 (C2/3/4/5), 53.1 (CH_3), 21.0, 20.8, 20.7 (CH_3C=O). HRMS (ESI) for $C_{27}H_{28}NO_{12}$ $[M+NH_4]^+$: *m/z* calcd 558.1606; measured: 558.1610.

1-Hydroxyanthraquinone β-D-glucopyranuronide sodium salt **20**.

Compound **19** (21.24 g) was dissolved in acetone (230 mL) and 1.13 M NaOH (118 mL) added. After 5 min, pre-washed Amberlite IR 120 H^+ was added to the mixture, followed by charcoal. This was stirred for 5 min and then filtered through celite, washing with acetone/water (2:1). The filtrate was cooled to 4 °C for 2 h and the solid collected by filtration, washing sparingly with acetone to give a yellow solid (9.89 g). This was dissolved in water (35 mL), then charcoal was added, the mixture stirred for 5 min, and then through pre-washed celite. Acetone (90 mL) was added to the filtrate and the resulting solid collected by filtration to give compound **20** (3.37 g, 20.4%) as a yellow solid. m.p. 253.0-255.0 °C. Rotation. $[\alpha]_D^{24}$ - 82° (*c* 0.5 in DMSO). ^{1}H-NMR: (D_2O) δ 8.07 (2H, t, *J*=6.9 Hz, 2 x Ar-H), 7.88-7.77 (4H, m, 4 x Ar-H), 7.69 (1H, d, *J*=8.2 Hz, 1 x Ar-H), 5.06 (1H, d, *J*=7.3 Hz, H-1), 3.54 (1H, d, *J*=9.6 Hz, 1 x H), 3.42-3.19 (3H, m, 3 x H). ^{13}C NMR: (D_2O) δ 183.1, 182.2 (*C*=O 1-hydroxyanthraquinone), 172.1 (C-6), 158.5, 135.9, 135.2 (2 x Ar-C), 135.1, 134.3, 132.6, 127.3, 126.7,

123.5, 122.0, 121.0, 101.5 (C-1), 77.1, 74.1, 73.8, 72.5 (C-2/3/4/5). HRMS (ESI) for $C_{20}H_{15}O_9$ [M-Na]$^-$: *m/z* calcd 399.0722; measured: 399.0724.

1-Hydroxyanthraquinone 2′,3′,5′-tri-*O*-acetyl-β-D-ribofuranoside **21**.

A mixture of 1-hydroxyanthraquinone **16** (10 g, 44.60 mmol), 3 Å molecular sieves (30 g) and boron trifluoride etherate (0.74 mL, 6 mmol) was stirred in DCM (278 mL). To this, a solution of 1-trichloroacetimidyl-2,3,5-triacetyl-β-D-ribofuranose (44.4 g, 105.6 mmol) in DCM (181 mL) was added drop-wise over 11 minutes. The reaction was stirred at RT for 18 h before neutralising with triethylamine (0.74 mL, 5.3 mmol). The reaction was filtered through pre-washed celite to remove the molecular sieves, washing with DCM. The filtrate was concentrated under reduced pressure to an amber/brown solid. The residue was triturated in methanol (100 mL) and cooled for 1 h. The solid was collected by filtration, and washed with methanol to give an orange solid (8.92 g), however TLC showed the solid was unreacted starting material. The filtrate from the obtained solid was concentrated under reduced pressure to a brown oil (45.55 g). The oil was triturated four times using DI water (50 mL) containing AcOH (0.15 mL) before dissolving in DCM (150 mL). The organic solution was washed with DI water (1 x 150 mL), then 0.5M NaOH (1 x 150 mL), then DI water (2 x 200 mL), before drying over $MgSO_4$ and concentrated under reduced pressure to a brown oil (23.03 g). The oil contained residual sugar, and so the trituration and washing steps were repeated to yield a brown oil (20.13g). This was purified by flash chromatography using C_{60} silica gel (900 g), eluting with toluene/acetone (20:1) collecting fractions of approx. 200 mL. Fractions 34-40 were combined and concentrated under reduced pressure to give an orange oil (3.48 g). The oil was triturated in IMS (7 mL) and cooled at -20 °C for 1 h. The crude product was collected by filtration and washed with IMS to give a yellow solid (400 mg). This purified further by recrystallisation from IMS, and the resulting solid collected by filtration to give compound **21** (370mg, 1.72%) as a yellow solid. m.p. 142.9-144.1 °C. ^{1}H-NMR: (DMSO-d_6) δ 8.17 (2H, t, *J*=7.3 Hz, 2 x Ar-H), 7.95-7.84 (4H, m, 4 x Ar-H), 7.66 (1H, d, *J*=8.2 Hz, 1 x Ar-H), 6.12 (1H, s, H-1), 5.59-5.55 (2H, m, H-2/3), 4.46-4.30 (2H, m, H-5a/5b), 4.05 (1H, dd, *J*=12.4 Hz, *J*=6.0 Hz, H-4), 2.15 (3H, s, C*H*$_3$C=O), 2.09 (3H, s, C*H*$_3$C=O), 1.80 (3H, s, C*H*$_3$C=O). HRMS (ESI) for $C_{25}H_{26}NO_{10}$ [M+NH$_4$]$^+$: *m/z* calcd 500.1551; measured: 500.1554.

1-Hydroxyanthraquinone β-D-ribofuranoside **22**.

Compound **21** (250 mg, 0.52 mmol) was suspended in methanol (1 mL). NaOMe solution in methanol (2.17M, 0.1 mL) was added and the reaction mixture stirred in the dark for 0.5 h. The resulting solid was collected by filtration, washed with a small amount of methanol to afford compound **22** as a yellow powder (150 mg, 81.2%). m.p. 182.0-183.6 °C. ^{1}H-NMR: (DMSO-d_6) δ 8.09 (2H, d, *J*=7.8 Hz, 2 x Ar-H), 7.88-7.76 (4H, m, 4 x Ar-H), 7.62 (1H, d, *J*=8.2 Hz, Ar-H), 5.66 (1H, s, H-1), 5.41 (1H, d, *J*=4.1 Hz, OH), 5.09 (1H, d, *J*=6.9 Hz, OH), 4.61 (1H, t, *J*=5.3 Hz, OH), 4.22-4.14 (2H, m, H-2/3), 3.93-3.88 (1H, m, H-4), 3.56-3.51 (1H, m, H-5a), 3.31-3.25 (1H, m, H-5b). ^{13}C NMR: (DMSO-d_6) δ 183.2, 181.7, 157.3, 135.6, 135.5, 135.1, 135.0, 134.2, 132.5, 127.2, 126.7, 123.6, 122.1, 120.8, 105.8 (C-1), 85.2, 75.1, 71.1 (C-2/3/4), 63.4 (C-5). HRMS (ESI) for $C_{19}H_{16}ClO_7$ [M+Cl]$^-$: *m/z* calcd 391.0590; measured: 391.0593.

Table S1. Comparison of substrate **18** and X-Gal for the detection of 40 strains of *E. coli.* Strains F1-F38 were obtained from faeces samples.

	Strain	Control (no substrate)		1-Hydroxyanthraquinone β-D-galactopyranoside **18** (300 mg L^{-1})*		5-Bromo-4-chloro-3-indolyl-β-D-galactopyranoside (X-Gal) (80 mg L^{-1})	
	Reference	Growth[a]	Colour[b]	Growth[a]	Colour[b]	Growth[a]	Colour[b]
1	*E. coli* (F1)	+	-	+	+ yellow	+	+green
2	*E. coli* (F2)	+	-	+	+ yellow	+	+green
3	*E. coli* (F3)	+	-	+	+ yellow	+	+green
4	*E. coli* (F4)	+	-	+	+ yellow	+	+green
5	*E. coli* (F5)	+	-	+	+ yellow	+	+green
6	*E. coli* (F6)	+	-	+	+ yellow	+	+green
7	*E. coli* (F7)	+	-	+	+ yellow	+	+green
8	*E. coli* (F8)	+	-	+	+ yellow	+	+green
9	*E. coli* (F9)	+	-	+	+ yellow	+	+green
10	*E. coli* (F10)	+	-	+	+ yellow	+	+green
11	*E. coli* (F11)	+	-	+	+ yellow	+	+green
12	*E. coli* (F12)	+	-	+	+ yellow	+	+green
13	*E. coli* (F13)	+	-	+	+ yellow	+	+green
14	*E. coli* (F14)	+	-	+	+ yellow	+	+green
15	*E. coli* (F15)	+	-	+	+ yellow	+	+green
16	*E. coli* (F16)	+	-	+	+ yellow	+	+green
17	*E. coli* (F17)	+	-	+	+ yellow	+	+green
18	*E. coli* (F18)	+	-	+	+ yellow	+	+green
19	*E. coli* (F19)	+	-	+	+ yellow	+	+green
20	*E. coli* (F20)	+	-	+	+ yellow	+	+green
21	*E. coli* (F21)	+	-	+	+ yellow	+	+green
22	*E. coli* (F22)	+	-	+	+ yellow	+	+green
23	*E. coli* (F23)	+	-	+	+ yellow	+	+green
24	*E. coli* (F24)	+	-	+	+ yellow	+	+green
25	*E. coli* (F25)	+	-	+	+ yellow	+	+green
26	*E. coli* (F26)	+	-	+	+ yellow	+	+green
27	*E. coli* (F27)	+	-	+	+ yellow	+	+green
28	*E. coli* (F28)	+	-	+	+ yellow	+	+green
29	*E. coli* (F29)	+	-	+	+ yellow	+	+green
30	*E. coli* (F30)	+	-	+	+ yellow	+	+green
31	*E. coli* (F31)	+	-	+	+ yellow	+	+green
32	*E. coli* (F32)	+	-	+	+ yellow	+	+green
33	*E. coli* (F33)	+	-	+	+ yellow	+	+green
34	*E. coli* (F34)	+	-	+	+ yellow	+	+green
35	*E. coli* (F35)	+	-	+	+ yellow	+	+green
36	*E. coli* (F36)	+	-	+	+ yellow	+	+green
37	*E. coli* (F37)	+	-	+	+ yellow	+	+green
38	*E. coli* (F38)	+	-	+	+ yellow	+	+green
39	NCTC 10418	+	-	+	+ yellow	+	+green
40	NCTC 12241	+	-	+	+ yellow	+	+green

*Results with 100 mg L^{-1} were identical with less intense yellow colour (data not shown).

[a] + good growth, - no growth.

[b] + strong colour.

Table S2. Comparison of substrate **18** and X-Gal for the detection of 12 strains of *Enterobacterales*.

			1-Hydroxy-anthraquinone β-D-galactopyranoside **18** (300 mg L^{-1})		1-Hydroxy-anthraquinone β-D-galactopyranoside **18** (100 mg L^{-1})		5-Bromo-4-chloro-3-indolyl-β-D-galactopyranoside (X-Gal) (80 mg L^{-1})	
	Strain	Reference	Growth[a]	Colour[b]	Growth[a]	Colour[b]	Growth[a]	Colour[b]
1	*Citrobacter freundii*	NCTC 9750	+	+ yellow	+	+ yellow	+	+ green
2	*Cronobacter sakazakii*	NCTC 11467	+	+ yellow	+	+ yellow	+	+ green
3	*Hafnia alvei*	NCTC 6578	-	-	-	-	+	+ green
4	*Klebsiella ozaenae*	NCTC 9601	+	+ yellow	+	+ yellow	+	+ green
5	*Klebsiella pneumoniae*	NCTC 10896	+	+ yellow	+	+ yellow	+	+ green
6	*Pantoea agglomerans*	NCTC 9381	+	+ yellow	+	+ yellow	+	+ green
7	*Providencia stuartii*	NCTC 10318	+	-	+	-	+	-
8	*Serratia ficaria*	NCTC 12148	+	+ yellow	+	+ yellow	+	+ green
9	*Serratia fonticola*	NCTC 12147	+	+ yellow	+	+ yellow	+	+ green
10	*Shigella flexneri*	NCTC 8192	+	-	+	-	+	-
11	*Shigella sonnei*	NCTC 8586	+	+ yellow	+	+ yellow	+	+ green
12	*Morganella morganii*	NCTC 232	+	-	+	-	+	-

All strains showed good growth and no coloration in medium without substrate added (data not shown).

[a] + good growth, - no growth.

[b] + strong colour, - no colour.

Table S3. Comparison of substrate **20** and X-Glucuronide for the detection of 40 strains of *E. coli.* Strains F1-F38 were obtained from faeces samples.

	Strain	Control (no substrate)		1-Hydroxy-anthraquinone β-D-glucopyranuronide sodium salt **20** (300 mg L^{-1})		1-Hydroxy-anthraquinone β-D-glucopyranuronide sodium salt **20** (100 mg L^{-1})		5-Bromo-4-chloro-3-indolyl-β-D-glucopyranuronide sodium salt (X-Glucuronide) (80 mg L^{-1})	
	Reference	Growth[a]	Colour[b]	Growth[a]	Colour[b]	Growth[a]	Colour[b]	Growth[a]	Colour[b]
1	E. coli (F1)	+	-	+	-	+	-	+	+green
2	E. coli (F2)	+	-	+	-	+	-	+	+green
3	E. coli (F3)	+	-	+	+ yellow	+	+/- yellow	+	+green
4	E. coli (F4)	+	-	+	+ yellow	+	+/- yellow	+	+green
5	E. coli (F5)	+	-	+	+ yellow	+	+/- yellow	+	+green
6	E. coli (F6)	+	-	+	-	+	-	+	-
7	E. coli (F7)	+	-	+	-	+	-	+	-
8	E. coli (F8)	+	-	+	+ yellow	+	+/- yellow	+	+green
9	E. coli (F9)	+	-	+	-	+	-	+	-
10	E. coli (F10)	+	-	+	-	+	-	+	+green
11	E. coli (F11)	+	-	+	+ yellow	+	+/- yellow	+	+green
12	E. coli (F12)	+	-	+	-	+	-	+	+green
13	E. coli (F13)	+	-	+	+ yellow	+	+/- yellow	+	+green
14	E. coli (F14)	+	-	+	+ yellow	+	-	+	+green
15	E. coli (F15)	+	-	+	+ yellow	+	-	+	+green
16	E. coli (F16)	+	-	+	+ yellow	+	-	+	+green
17	E. coli (F17)	+	-	+	-	+	-	+	+green
18	E. coli (F18)	+	-	+	+ yellow	+	+/- yellow	+	+green
19	E. coli (F19)	+	-	+	-	+	-	+	+green
20	E. coli (F20)	+	-	+	+ yellow	+	+/- yellow	+	+green
21	E. coli (F21)	+	-	+	+/- yellow	+	-	+	+green
22	E. coli (F22)	+	-	+	-	+	-	+	+green
23	E. coli (F23)	+	-	+	+ yellow	+	+/- yellow	+	+green
24	E. coli (F24)	+	-	+	-	+	-	+	-
25	E. coli (F25)	+	-	+	+ yellow	+	+/- yellow	+	+green
26	E. coli (F26)	+	-	+	-	+	-	+	+green
27	E. coli (F27)	+	-	+	+ yellow	+	+/- yellow	+	+green
28	E. coli (F28)	+	-	+	+ yellow	+	+/- yellow	+	+green
29	E. coli (F29)	+	-	+	+ yellow	+	+/- yellow	+	+green
30	E. coli (F30)	+	-	+	-	+	-	+	-
31	E. coli (F31)	+	-	+	-	+	-	+	-
32	E. coli (F32)	+	-	+	+/- yellow	+	-	+	+green
33	E. coli (F33)	+	-	+	-	+	-	+	-
34	E. coli (F34)	+	-	+	+ yellow	+	+/- yellow	+	+green
35	E. coli (F35)	+	-	+	+ yellow	+	+/- yellow	+	+green
36	E. coli (F36)	+	-	+	-	+	-	+	+green
37	E. coli (F37)	+	-	+	+ yellow	+	+/- yellow	+	+green
38	E. coli (F38)	+	-	+	-	+	-	+	+green
39	NCTC 10418	+	-	+	+ yellow	+	+/- yellow	+	+green
40	NCTC 12241	+	-	+	+ yellow	+	+/- yellow	+	+green

[a] + good growth, - no growth.

[b] + strong colour, +/- weak colour, - no colour.
